# Bacteremia caused by *Desulfovibrio desulfuricans* with the intestinal tract as the portal of entry: two case reports and a literature review

**DOI:** 10.1186/s12879-024-09623-3

**Published:** 2024-07-24

**Authors:** Kotone Yamaizumi, Moe Kyotani, Tsuneaki Kenzaka

**Affiliations:** 1 Department of Internal Medicine, Hyogo Prefectural Tamba Medical Center, 2002-7 Iso, Hikami-cho, Tamba, Hyogo 669-3495 Japan; 2 Division of Community Medicine and Career Development, Kobe University Graduate School of Medicine, 2-1-5, Arata-cho, Hyogo-ku, Kobe, Hyogo 652-0032 Japan

**Keywords:** Abdominal symptoms, Case report, *Desulfovibrio desulfuricans*, Gastrointestinal complications, Older population

## Abstract

**Background:**

*Desulfovibrio desulfuricans* (*D. desulfuricans*), a commensal anaerobic gram-negative rod endemic to the soil environment and human gastrointestinal tract, rarely causes bloodstream infections. We report two rare cases of bacteremia caused by *D. desulfuricans* in which the intestinal tract was the portal of entry. In addition, we summarize findings on *D. desulfuricans*.

**Case presentation:**

Case 1: A 51-year-old man presented to the emergency department with the chief complaints of fever and right lower abdominal pain. He was admitted to the hospital with ascending colonic diverticulitis and received empirical antibacterial therapy with piperacillin/tazobactam. Blood culture revealed *D. desulfuricans*. The patient was discharged after 2 weeks of antimicrobial therapy. Case 2: A 95-year-old woman presented to our hospital with a chief complaint of fever. Owing to an elevated inflammatory response and pyuria, the patient was diagnosed with pyelonephritis and treated with ceftriaxone. *Klebsiella pneumoniae* was detected in her urine culture, while *D. desulfuricans* was detected in her blood culture. The patient was then treated with ampicillin/sulbactam for 14 days. The fecal occult blood test result was positive, suggesting a colonic mucosal lesion, such as a malignant tumor, may have been the portal of entry for *D. desulfuricans* bacteremia. Previous literature reviews indicate that *D. desulfuricans* bacteremia often results from liver or renal abscesses, intestinal lesions, among others, serving as the portal of entry. Although no specific underlying disease has been reported, it is more common in the older population. We encountered two cases of *D. desulfuricans* bacteremia and combined them with 15 cases from previous studies to explore the characteristics of the disease. The proportion of patients aged $\:\ge\:$60 years was 73.7%; overall, 73.7% had gastrointestinal complications, and 63.2% had abdominal symptoms at the time of presentation.

**Conclusions:**

We encountered two rare cases of *D. desulfurican* bacteremia. This type of bacteremia is more common in elderly people over 60 years of age and is often associated with hepatobiliary and gastrointestinal diseases.

## Background

*Desulfovibrio* is a genus of commensal anaerobic Gram-negative rods that are ubiquitous to the soil environment and human gastrointestinal tract. These bacteria have a characteristic curved morphology and motility, requiring more time for development than usual [1, 2].

The genus *Desulfovibrio* currently includes more than 60 species, of which six species, *Desulfuricans*,* Desulfuricans fairfieldensis*,* Desulfuri vulgaris*,* Desulfuri piger*,* Desulfuri legallii*, and *Desulfuri intestinalis* are known to be pathogenic to humans [3]. *D. desulfuricans* produces hydrogen sulfide, which has a characteristic sulfur-like odor. The first human infection, a bloodstream infection associated with liver abscesses, was reported by Tee et al. in 1996 [4]. Although the report by Porschen et al. in 1977 was considered the first [5], it is highly likely that it was caused by *D. fairfieldensis* and not *D. desulfuricans* based on the characteristics of the bacteriological examination [6]. A bloodstream infection by *D. desulfuricans* is often caused by intra-abdominal abscesses such as liver abscesses or intestinal lesions acting as a portal of entry [7, 8].

However, there are few reports of infections caused by *D. desulfuricans*, especially those involving the bloodstream [1, 4, 7, 8]. Here, we report two rare cases of bacteremia caused by *D. desulfuricans* entering the intestinal tract. We also summarize the previous literature on *D. desulfuricans* bacteremia.

## Case presentation

### Case 1

A 51-year-old man with independent activities of daily living was admitted to our hospital with the chief complaints of fever and right lower abdominal pain. He had a history of diverticulitis at the age of 30 years. He was taking amlodipine 5 mg/day for hypertension.

The day before his visit, he developed severe right lower abdominal pain after lunch and was treated with 60 mg loxoprofen. On the evening of the same day, he developed a fever of 38.6 ℃. Because his lower abdominal pain did not improve on the day of his visit, he visited the emergency room of our hospital. At admission, the patient’s vital signs were as follows: clear consciousness, a respiratory rate of 16 breaths/min, a temperature of 38.2 °C, a pulse of 105 beats/min, a blood pressure of 179/105 mmHg, and a peripheral oxygen saturation (SpO_2_) of 99% (in room air). Physical examination of the patient revealed no increase or decrease in intestinal peristalsis. Tenderness and tapping pain were observed from the right side of the umbilicus to the right lower abdomen. Recoil pain was noted as a symptom of peritoneal irritation; however, there was no evidence of muscular defense or Murphy’s sign.

**Fig. 1:**
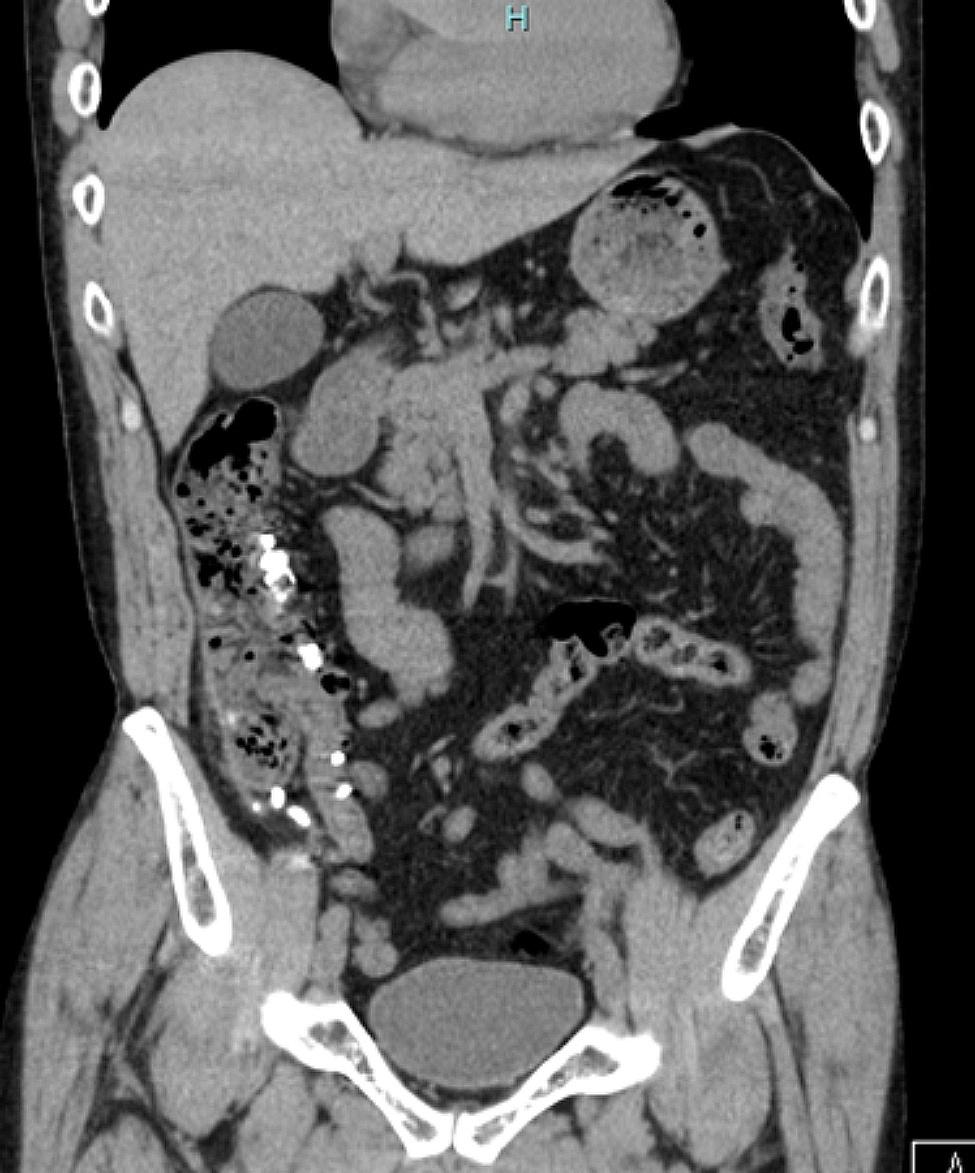
A non-contrast CT scan of the abdomen in case 1 CT shows multiple highly absorptive diverticula in the ascending colon, some with free air. CT, computed tomography

The patient’s white blood cell count was 16,700/µL, and his C-reactive protein (CRP) level was 0.21 mg/dL. No abnormalities on urinalysis were found. A non-contrast computed tomography (CT) scan of the abdomen showed multiple highly absorptive diverticula in the ascending colon, some with free air (Fig. 1).

Based on these findings, the patient was diagnosed with ascending colonic diverticulitis. The patient was hospitalized and treated with intravenous piperacillin/tazobactam (4.5 g every 6 h). Subsequently, the CRP levels peaked at 13.4 mg/dL on day 4 of hospitalization and improved thereafter. His fever and abdominal pain gradually improved and resolved by day 6. On day 7 of hospitalization, two sets of blood cultures obtained on day 1 of hospitalization revealed the development of a spiral-shaped gram-negative rod. Bacterial identification by mass spectrometry identified the organism as *D. desulfuricans*. The patient was on adequate antimicrobial therapy, thus repeat blood cultures were not performed.

The patient was diagnosed with *D. desulfuricans* bacteremia associated with ascending colonic diverticulitis. The patient was discharged from hospital after 2 weeks of antimicrobial therapy. Three years have passed since the time of discharge without any recurrence.

### Case 2

A 95-year-old woman presented to our hospital with a chief complaint of fever. She required nursing care for almost all her personal needs, including eating and toileting. She had previously been diagnosed with pancytopenia. However, since she declined further testing, a search for the cause of pancytopenia was not conducted. On the day of her visit, she presented to our emergency room because of cyanosis and a fever of 38.5 °C. Her medical history included pancytopenia of an unknown etiology. The patient also had aortic stenosis, bronchial asthma, hypertension, and dementia.

On arrival, her vital signs were clear, with a blood pressure was 112/77 mmHg, an irregular pulse of 64 beats/min, a temperature of 36.9℃, a respiratory rate of 12 breaths/min, and an SpO_2_ of 99% (in room air).

Physical examination revealed pallor of the eyelid and conjunctiva. Hemorrhagic spots were observed in the oral mucosa. Course crackles were auscultated in the right middle and lower lung fields, and tenderness was elicited by tapping over the right costovertebral angle. An ejection systolic murmur (Levine III/VI) was auscultated, with the strongest point in the second intercostal space of the right sternal border.

The patient’s white blood cell count was 6380/µL, and her CRP level was 8.1 mg/dL. Urinalysis showed a leukocyte count of > 100/high-power field. Simple chest radiography showed no evidence of pneumonia, and simple abdominal CT revealed no enlarged renal pelvis, ascites, or intestinal lesions. The patient was diagnosed with acute pyelonephritis based on the presence of fever and urinalysis findings. The patient was hospitalized and treated with antimicrobial therapy with ceftriaxone (1 g every 24 h). Her CRP level and fever gradually improved, with a peak CRP level observed on day 4 of hospitalization. *Klebsiella pneumoniae* was detected in her urine culture taken at initial visit. Furthermore, blood cultures on the third day of hospitalization revealed Gram-negative rods in two sets of blood culture, indicating bacteremia. Gram-negative rods were suspected to be *Fusobacterium*, and the antimicrobials were changed from ceftriaxone to ampicillin/sulbactam (3 g every 6 h). The Gram-negative rods that developed in both sets of blood cultures were subjected to mass spectrometry and identified as *D. desulfuricans*. At follow-up, two sets of repeat blood culture were negative because the patient was on antimicrobial therapy. Antimicrobial therapy was completed within 2 weeks, and the patient was discharged 35 days after rehabilitation. The final diagnosis was acute pyelonephritis due to *K. pneumoniae* and *D. desulfuricans* bacteremia.

*K. pneumoniae* was detected in the urine culture, whereas *D. desulfuricans* was detected in the blood culture. As they were different strains, a fecal occult blood test was performed; the test result was positive. Epigastroscopy and colonoscopy were proposed, although the patient refused to undergo these procedures. Two years have passed since discharge from the hospital, without any recurrence.

Table 1 shows the antimicrobial susceptibility results for the main antimicrobial agents in cases 1 and 2.

**Table 1:**
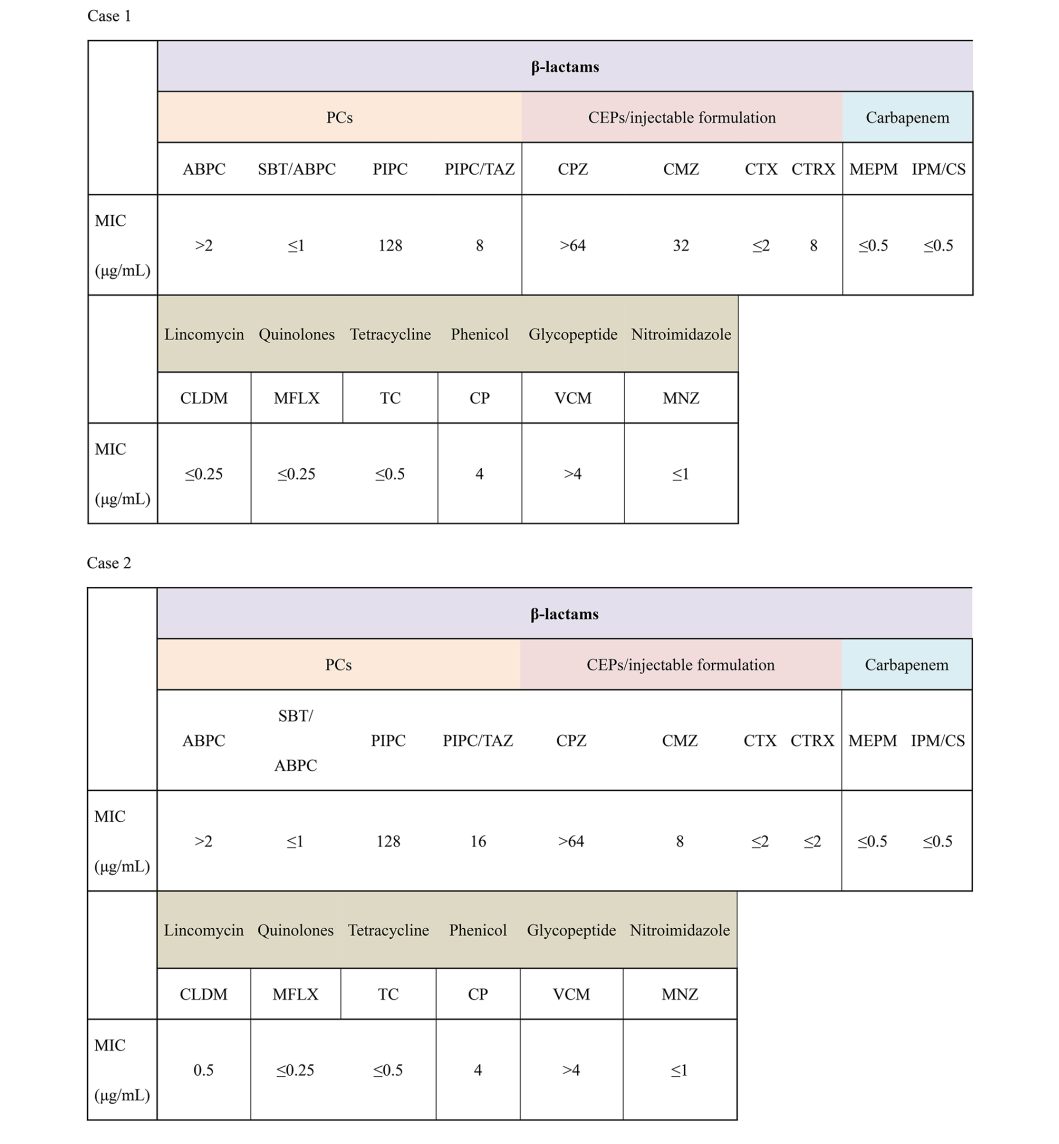
Antimicrobial susceptibility testing of the *D. desulfuricans* isolate case 1. The susceptibility categories were based on the Clinical and Laboratory Standards Institute (CLSI) classification. ABPC, ampicillin; ABPC/SBT, ampicillin sulbactam; CLDM, clindamycin; CMZ, cefmetazole; CP, chloramphenicol; CPZ, cefoperazon; CTRX, ceftriaxone; CTX, cefotaxime; IPM/CS, imipenem cilastatin; MEPM, meropenem; MFLX, moxifloxacin; MNZ, metronidazole; PIPC, piperacillin; PIPC/TAZ, piperacillin/tazobactam; TC, achromycin; VCM, vamcomycin

## Discussion

We present two rare cases of *D. desulfuricans* bacteremia, summarize previous reports of *D. desulfuricans* bacteremia using PubMed, and describe their clinical features.

A PubMed search for “*Desulfovibrio desulfuricans*” and “bacteremia” yielded 17 previously reported cases. Table 2 shows our two cases, in addition to those previously reported [1, 4, 7–21]. The median age of the patients was 69 (range, 18–95) years, 14 (73.7%) patients were aged $\:\ge\:$60 years, and the male-to-female ratio was 10:9. The outcome was death in two cases [15, 16], corresponding to a mortality rate of 10.5%. Twelve (63.2%) patients presented with fever [1, 4, 8, 9, 13, 16–20], and 12 (63.2%) had abdominal symptoms [1, 8–10, 13, 15, 16, 18–21].

**Table 2:** Reports of *D. desulfuricans* bacteremia

Case	Author	Reference number	Country	Age	Sex	Fever	Abdomen symptoms	Antibacterial drugs	Underlying disease	Mixed infection	Diagnostic method	Outcome
1	Lopez Alonso et al.	9	Spain	18	Female	+	+	AMPC/CVA	Tetralogy of Fallot, Necrotizing appendicitis	Poly/*Christensenella minuta*	16 S rRNA	Survived
2	Machaca et al.	10	Argentina	30	Female	Unknown	+	PIPC/TAZ	Necrotizing appendicitis	Poly/*Escherichia coli*	MS	Survived
3	Nasreddine et al.	8	Belgium	53	Male	+	+	AMPC/CVA	None	Mono	MS	Survived
4	Marquis et al.	11	America	53	Female	-	−	MNZ	Type 2 diabetes mellitus, Inflammatory bowel disease	Mono	MS	Survived
5	Tanamachi et al.	12	Japan	60	Male	Unknown	Unknown	CTRX + EM	Chronic kidney disease	Mono	Biochemical	Survived
6	Goldstein et al.	1	America	64	Male	+	+	DOXY	Diarrhea after international travel	Mono	16 S rRNA	Survived
7	Verstreken et al.	13	Belgium	69	Female	+	+	CAM	Type 2 diabetes mellitus, PCS after liver transplant, Repeated cholangitis, Ulcerative colitis	Mono	16 S rRNA	Survived
8	Otto et al.	14	France	69	Male	Unknown	Unknown	OFLX + PIPC/TAZ	Postoperative recurrence of colon cancer	Mono	16 S rRNA	Survived
9	Fernández Vecilla D et al.	15	Spain	69	Male	-	+	MEPM + CLDM + DAP	Fournier gangrene, untreated chronic obstructive pulmonary disease	Poly/*Escherichia coli*	MS	Died
10	Silvia C Predari et al.	16	Argentina	75	Female	+	+	CTRX	Post total hysterectomy, uterine sarcoma	Mono	16 S rRNA	Died
11	Yamazaki et al.	17	Japan	83	Female	+	Unknown	ABPC/CVA	Liver abscess	Mono	16 S rRNA	Survived
12	Tee et al.	4	Unknown	82	Female	+	−	CPFX + MNZ	Chronic diarrhea, post cholecystectomy	Mono	16 S rRNA	Survived
13	Koyano et al.	18	Japan	82	Male	+	+	AMPC/CVA	Gallbladder cholelithiasis	Poly/*Eggerthella lenta*	16 S rRNA	Survived
14	Fujiwara et al.	19	Japan	84	Male	+	+	AMPC/SBT	endovascular aortic repair (EVAR), hypertension, type 2 DM, and chronic kidney disease	Mono	16s rRNA	Survived
15	Liderot et al.	20	Sweden	86	Female	+	+	AMPC	Pressure ulcer near the anal canal	Mono	16 S rRNA	Survived
16	Hagiwara et al.	21	Japan	87	Male	-	+	CZOP + VCM	Type 2 DM, after treatment of aspiration pneumonia	Mono	16 S rRNA	Survived
17	Hagiya et al.	7	Japan	88	Male	Unknown	Unknown	MNZ	Thoracic aortic aneurysm, post TEVAR	Mono	16 S rRNA	Survived
18		Case 1	Japan	51	Male	+	+	PIPC/TAZ	Diverticulitis, Multiple diverticula	Mono	MS	Survived
19		Case 2	Japan	95	Female	+	−	ABPC/SBT	Pancytopenia, Positive fecal occult blood	Mono	MS	Survived

ABPC/SBT, ampicillin/sulbactam; AMPC/CVA, ampicillin/clavulanic acid; CAM, clarithromycin; CLDM, clindamycin; CPFX, ciprofloxacin; CZOP, cefozopran; DAP, daptomycin; DOXY, doxycycline; MEPM, meropenem; MS, mass spectrometry; MNZ, metronidazole; OFLX, ofloxacin; PIPC/TAZ, piperacillin/tazobactam; VCM, vancomycin

Four (21.1%) cases involved mixed infections with enterobacteria, including *Eggerthella lenta*, *Christensenella minuta*, and *Escherichia coli*. According to previous reports, older age (> 60 years) is a risk factor, and fragility of the gastrointestinal mucosa due to aging may be a contributing factor [7, 8]. Fourteen of the 19 (73.7%) patients had a gastrointestinal disease [1, 4, 9–11, 13–15, 17–20], while the remaining did not.

As in case 1, reports of bloodstream infections in patients aged < 60 years were rare, occurring in only 3 of 19 cases [9, 10].

In case 1, diverticulitis occurred, and the intestinal tract was considered the portal of entry. In case 2, there was no underlying lesion, but the different causative organisms of urinary tract infection and bacteremia, along with the positive result in fecal occult blood test, suggested the possibility of a malignant tumor or colonic mucosal lesion (e.g. inflammatory colonic lesion) as the entry point. However, a diagnosis was not made as the patient did not wish to undergo an endoscopic examination.

*D. desulfuricans* comprises two genotypes, Essex 6 and MB [2, 21]. The characteristics of their antimicrobial susceptibilities and the susceptibilities of cases 1 and 2 are shown in Table 2. Essex 6 isolates are less susceptible to β-lactams than MB isolates and are highly resistant to both piperacillin (PIPC) and cefoxitin (CFX) [2]. On the other hand, MB isolates are moderately susceptible to PIPC and CFX [2]. Based on a comparison of antimicrobial susceptibilities, the microbial agents detected in cases 1 and 2 were assumed to be *D. desulfuricans* MB. There is no established antimicrobial regimen for use against *D. desulfuricans*, and the optimal antimicrobial therapy remains unknown. In situations where the empirical use of broad-spectrum antimicrobial agents fails or when helminths are identified in anaerobic cultures, these organisms should be kept in mind and identified at the species level using mass spectrometry or 16 S rRNA gene sequence analysis. Strain identification may guide more optimal antimicrobial selection. Moreover, eight cases were reported from Japan, which may have been due to geographical factors, although the precise reason remains unclear. Further studies are anticipated in the future regarding this relationship.

## Conclusion

We encountered two rare cases of *D. desulfurican* bacteremia and analyzed them along with 17 previous cases from the literature to characterize this condition. The proportion of patients aged ≥ 60 years was 73.7%; overall, 73.7% had gastrointestinal complications, and 63.2% had abdominal symptoms at the time of hospital admission.

## Data Availability

All data generated or analyzed during this study are included in this published article.

## Abbreviations

CFX: Cefoxitin
CRP: C-reactive protein
CT: Computed tomography
D. desulfuricans: Desulfovibrio desulfuricans
PIPC: Piperacillin
SpO_2_: Peripheral oxygen saturation
